# The hospital microbiome project: meeting report for the UK science and innovation network UK-USA workshop ‘beating the superbugs: hospital microbiome studies for tackling antimicrobial resistance’, October 14th 2013

**DOI:** 10.1186/1944-3277-9-12

**Published:** 2014-12-08

**Authors:** Jack Westwood, Matthew Burnett, David Spratt, Michael Ball, Daniel J Wilson, Sally Wellsteed, David Cleary, Andy Green, Emma Hutley, Anna Cichowska, Susan Hopkins, Mark Wilcox, Anthony Kessel, Ghada Zoubiane, Lara Bethke, Derrick W Crook, Jimmy Walker, Mark Sutton, Philip Marsh, Ginny Moore, Peter Wilson, Alison Holmes, Peter Hoffman, Chris Smith, Beryl Oppenheim, Julian Parkhill, Neil Woodford, Julie Robotham, Claire Kidgell, Martin Anyim, Gabriella Gilkes, Dawn Field, Josh Quick, Tony Pickering, Benjamin C Kirkup, Jack Gilbert

**Affiliations:** 1 British Consulate-General, Chicago, IL 60611, USA; 2 The Naked Scientists, Department of Pathology, Cambridge University, Cambridge, UK; 3 Department of Microbial Diseases, UCL Eastman Dental Institute, London, UK; 4 Biotechnology and Biological Science Research Council, Polaris House, Swindon, UK; 5 Nuffield Department of Medicine, University of Oxford, John Radcliffe Hospital, Oxford, UK; 6 Department of Health, Richmond House, London, UK; 7 Defence Science and Technology Laboratory, Porton Down, Wiltshire, UK; 8 Royal Centre for Defence Medicine, Birmingham, UK; 9 Public Health England, London, UK; 10 University of Leeds & Leeds Teaching Hospitals, Leeds, UK; 11 Medical Research Council, London, UK; 12 The Wellcome Trust, London, UK; 13 Nuffield Department of Medicine, University of Oxford, John Radcliffe Hospital, Oxford OX3 9DU, UK; 14 Biosafety Unit, Public Health England, Porton Down, Salisbury, UK; 15 HPA Microbiology Services, Porton Down, Salisbury, UK; 16 Clinical Microbiology & Virology, UCLH NHS Foundation Trust, London, UK; 17 Commonwealth Building, Imperial College, London, UK; 18 Antimicrobial Resistance and Healthcare-Associated Infections Reference Unit, Public Health England, London, UK; 19 Addenbrooke’s Hospital Clinical, Microbiology and Public Health Laboratory, Cambridge, UK; 20 Queen Elizabeth Hospital Birmingham, Birmingham, UK; 21 Wellcome Trust Genome Campus, Wellcome Trust Sanger Institute, Hinxton, Cambridge, UK; 22 Modeling and Economics Unit, Statistics, Modeling and Economics Department, Public Health England, 61 Colindale Avenue, London, UK; 23 National Institute for Health Research, Evaluation, Trials and Studies Coordinating Centre (NETSCC), University of Southampton, Southampton, UK; 24 NIHR Invention for Innovation Programme, Richmond House, London, UK; 25 The Eden Project, Human Micro Biome Project, Cornwall PL24 2SG, UK; 26 NERC Centre for Ecology and Hydrology, Oxford, UK; 27 Institute of Microbiology and Infection, University of Birmingham, Birmingham, UK; 28 North West Lung Research Centre, University of South Manchester, Wythenshawe Hospital, Manchester, UK; 29 Department of Wound Infections, Walter Reed Army Institute of Research, Bethesda, MD, USA; 30 Department of Ecology and Evolution, University of Chicago, Chicago, IL, USA; 31 Institute for Genomic and Systems Biology, Argonne National Laboratory, Argonne, IL, USA

**Keywords:** Antibiotic resistance, Nosocomial infections, Hospital microbiome, Superbugs

## Abstract

The UK Science and Innovation Network UK-USA workshop ‘Beating the Superbugs: Hospital Microbiome Studies for tackling Antimicrobial Resistance’ was held on October 14th 2013 at the UK Department of Health, London. The workshop was designed to promote US-UK collaboration on hospital microbiome studies to add a new facet to our collective understanding of antimicrobial resistance. The assembled researchers debated the importance of the hospital microbial community in transmission of disease and as a reservoir for antimicrobial resistance genes, and discussed methodologies, hypotheses, and priorities. A number of complementary approaches were explored, although the importance of the built environment microbiome in disease transmission was not universally accepted. Current whole genome epidemiological methods are being pioneered in the UK and the benefits of moving to community analysis are not necessarily obvious to the pioneers; however, rapid progress in other areas of microbiology suggest to some researchers that hospital microbiome studies will be exceptionally fruitful even in the short term. Collaborative studies will recombine different strengths to tackle the international problems of antimicrobial resistance and hospital and healthcare associated infections.

## Introduction

Antimicrobial resistance (AMR) is a global challenge and costs the NHS an estimated £1 billion a year, affecting tens of thousands of lives [1]. UK leadership of the G8 in 2013 led to a joint statement from the G8 science ministers in June that identified AMR as a key priority [2]. A Department of Health/DEFRA 5 year strategy for addressing AMR coincided with publication of the Antibiotic Resistance Threat Report by the US Center for Disease Control and Prevention [3]. These publications set out ways for both countries to steward existing antibiotics and hasten the development of new antimicrobial chemotherapies. Meanwhile, one of the most important venues for transmission of antimicrobial resistant pathogens is in healthcare settings. Approximately 5% of all patients admitted to a medical facility will be diagnosed with a hospital-associated infection [4]. These infections cost an estimated 36–45 billion dollars annually (2007 dollars [5]) and result in approximately 100,000 deaths [6].

Through the UK Research Councils, the Wellcome Trust and National Institute for Health Research (NIHR), the UK has invested heavily in transmission studies of microbes that pose the biggest threat, namely meticillin-resistant *Staphylococcus aureus* (MRSA) and *Clostridium difficile*. These studies are particularly prominent for their advanced use of whole genome sequencing in the service of epidemiology and infection control. The same advances in sequencing technology which enable whole genome epidemiology also enable the use of phylogenetically open microbial community characterization and the high-throughput molecular detection of antimicrobial resistance genes, representing an orthogonal approach to hospital microbial ecology. The Hospital Microbiome Project [7], led from the University of Chicago, the Walter Reed Army Institute of Research and the Uniformed Services University of the Health Sciences, brings these technologies together in a community ecology approach to the hospital, which is less directly connected to individually diagnosed infections and their control. This approach is a significant departure from existing whole genome strategies, which sensitively examine cultured clinical and environmental isolates of bacteria or viruses that present particular interest and generally threaten nosocomial outbreaks. Microbiome studies provide reduced characterization of individual strains while embracing a wider phylogenetic diversity from each sample.

Microbiome studies in hospitals seek to characterize the health-promoting and disease-causing bacteria in the hospital environment, to understand the consequences of cleaning, ventilation, sterilization, and prophylactic antibiotic use on the microbiome. The community of organisms within the hospital is important for several reasons. First, genes conferring antibiotic resistance reside in non-pathogenic organisms in even the least-likely abiotic environments [8]; certainly this is also true within hospital environments. These genes can be transferred to pathogens, thus resulting in new drug resistant, pathogenic organisms. Second, colonization resistance is the property of most microbial communities; its mechanism is slowly being better described [8,9] the colonization resistance of the hospital microbiome – intact, disturbed or engineered – may have a role in infection control. Current hospital cleaning practices and antibiotic treatments do not consider the microbiome, but concentrate on controlling target microorganisms. Perturbation or ablation of a hospital’s microbial ecology through such interventions may therefore reduce the competition experienced by pathogenic organisms and result in their increased proliferations and capacity to cause disease. Finally, the health promoting, or probiotic effect of certain organisms and combinations of organisms is well characterized in certain environments, such as the gut. Indeed, obesity has been shown to correlate with particular variants of the gut microbiome [10,11]. The effect of the microbiome on the health of a hospital building and its patients is currently unstudied and potentially significant.

The largest field component of the Hospital Microbiome Project (HMP) is designed to characterize the taxonomic composition of surface-, air-, water-, and human- associated microbial communities in the University of Chicago’s Center for Care and Discovery (CCD) [12]. The 1st HMP Workshop (June 7th-8th 2012 [13];) explored the initial sampling strategy and approach to building science measurements, and led to the development of a full proposal to the Alfred P. Sloan Foundation, and the creation of the Hospital Microbiome Consortium [14]. The 2nd HMP Workshop (January 15th 2013 [15];) was held immediately prior to the start of sampling in the CCD and was responsible for last minute changes to sample design and sample handling, as well as numerous other facets of the project implementation.

Here we present discussion and conclusions from the UK Science and Innovation Network’s UK-USA workshop ‘Beating the Superbugs: Hospital Microbiome Studies for tackling Antimicrobial Resistance’, which was held on October 14th 2013 at the UK Department of Health, London. This brought expertise from the Hospital Microbiome Project in the US over to the UK, via support from the UK Foreign and Commonwealth Office, to interact with experts on AMR, pathogen transmission, building science, public health, etc. and to determine what UK research groups could contribute to the built environment and human-associated microbiome research? This was also an excellent opportunity to identify critical concerns and valuable suggestions as to how the multiple research arms associated with the eradication of nosocomial infections could be better integrated. Finally, the workshop agenda was laid out to facilitate maximum discussion time regarding the value of microbiome research in hospital environments. The meeting occurred over one day, and the agenda consisted of 9 short presentations on the problem, the US experience, the UK research portfolio, and funding opportunities. This was followed by extensive discussion on funding opportunities and the value of doing this research in the UK, and extensive discussion on the value of this research agenda, and the potential issues that such projects must consider.

### Meeting Participants

**Martin Anyim**, Assistant Director, NIHR Invention for Innovation Programme, Richmond House, 79 Whitehall, London, SW1A 2NS.

**Michael Ball**, Biotechnology and Biological Science Research Council, Polaris House, Swindon, SN2 1UH.

**Lara Bethke,** The Wellcome Trust, London, N6 5HR, United Kingdom.

**Matthew Burnett**, The Naked Scientists, Dept. of Pathology, Cambridge University, Tennis Court Road, Cambridge, UK, CB2 1QP.

**Anna Cichowska**, Public Health England, 133–155 Waterloo Road, London, SE1 8UG.

**David Cleary**, Defence Science and Technology Laboratory, Porton Down, Salisbury, Wiltshire SP4 OJQ, UK.

**Derrick Crook**, Nuffield Department of Medicine, University of Oxford, John Radcliffe Hospital, Oxford, OX3 9DU, UK.

**Dawn Field**, NERC Centre for Ecology and Hydrology, United Kingdom, OX10 8BB.

**Jack Gilbert**, Argonne National Laboratory, USA; Department of Ecology and Evolution, University of Chicago, USA.

**Gabriella Gilkes**- The Eden Project, Human Micro Biome Project, Cornwall, PL24 2SG, UK.

**Andrew Green**, Royal Centre for Defence Medicine, Birmingham B15 2SQ.

**Peter Hoffman**, Consultant Clinical Scientist, Antimicrobial Resistance and Healthcare-associated Infections Reference Unit, Public Health England, 61 Colindale Avenue, London NW9 5EQ, UK.

**Alison Holmes**, Professor in Infectious Diseases, Commonwealth Building, Imperial College, Du Cane Road, London W12 0NN.

**Susan Hopkins**, Public Health England, 133–155 Waterloo Road, London, SE1 8UG.

**Emma Hutley**, Consultant Microbiologist, Royal Centre for Defence Medicine, ICT Building, Vincent Drive, Birmingham, B15 2SQ.

**Anthony Kessel**, Public Health England, Wellington House, 133–155 Waterloo Road, London, SE1 8UG.

**Claire Kidgell**, National Institute for Health Research, Evaluation, Trials and Studies Coordinating Centre (NETSCC), University of Southampton, Alpha House, Enterprise Road, Southampton SO16 7NS, UK.

**Benjamin Kirkup** PhD: Department of Wound Infections, Walter Reed Army Institute of Research, USA.

**Philip Marsh**, Microbiology Service, Public Health England, Porton Down, Salisbury, SP4 0JG, UK.

**Ginny Moore**, Biosafety Unit, Public Health England, Porton Down, Salisbury SP4 0JG, UK.

**Beryl Oppenheim**, Consultant Microbiologist, Queen Elizabeth Hospital Birmingham, Birmingham B15 2TH.

**Mark Pallen**, Division of Microbiology and Infection, Warwick Medical School, University of Warwick, Coventry CV4 7AL.

**Julian Parkhill**, Wellcome Trust Sanger Institute, Wellcome Trust Genome Campus, Hinxton, Cambridge, CB10 1SA, UK.

**Tony Pickering**, North West Lung Research Centre, University of South Manchester, Wythenshawe Hospital, Manchester M23 9LT.

**Josh Quick**, Institute of Microbiology and Infection, University of Birmingham, Edgbaston, Birmingham, West Midlands, B15 2TT.

**Julie Robotham**, Modeling and Economics Unit, Statistics, Modeling and Economics Department, Public Health England, 61 Colindale Avenue, London NW9 5EQ.

**Chris Smith**, Addenbrooke’s Hospital Clinical, Microbiology and Public Health Laboratory/The Naked Scientists, Dept. of Pathology, University of Cambridge, Tennis Court Road, Cambridge CB2 1QP.

**David Spratt**, Department of Microbial Diseases, UCL Eastman Dental Institute, 256 Gray’s Inn Road, London, WC1X 8LD.

**Mark Sutton**, Public Health England, HPA Microbiology Services, Porton Down, Salisbury, SP4 0JG, UK.

**Jimmy Walker**, Biosafety Unit, Public Health England, Porton Down, Salisbury SP4 0JG, UK.

**Ursula Wells**, Department of Health, Section Head, Research and Development Directorate, Department of Health, 79 Whitehall, SW1A 2NS.

**Sally Wellsteed**, Team Leader Infection Control, Department of Health, Richmond House, 79 Whitehall, London SW1A 2NS.

**Jack Westwood**, British Consulate-General 625 N. Michigan Ave, Chicago, IL 60611.

**Mark Wilcox**, University of Leeds & Leeds Teaching Hospitals.

**Daniel Wilson**, Nuffield Department of Medicine, University of Oxford, John Radcliffe Hospital, Oxford, OX3 9DU, UK.

**Peter Wilson**, Consultant Microbiologist, Clinical Microbiology & Virology, UCLH NHS Foundation Trust, 60 Whitfield Street, London W1T 4EU.

**Neil Woodford**, Head, Antimicrobial Resistance and Healthcare Associated Infections (AMRHAI) Reference Unit, Reference Microbiology Services, Public Health England, 61 Colindale Avenue, London NW9 5EQ.

**Ghada Zoubiane**, Programme Manager, Medical Research Council, One Kemble street, London WC2B 4AN.

### Session 1: introducing the problem, and the US and UK research

#### Moderated by Jack Westwood (Head of Science and Innovation, British Consulate-General, Chicago)

The UK Science and Innovation Network (SIN) together with Public Health England and the Alfred P Sloan Foundation (US) supported and convened a workshop in London on 14th October to bring together Principal Investigators from the HMP with 35 leading UK experts, policy-makers and funding agencies to discuss the scope for implementing microbiome studies in UK hospitals. The first session included an introduction from Jack Westwood who started off by thanking all for their attendance and explaining why the UK SIN was interested in promoting US-UK collaboration on hospital microbiology. He also highlighted the importance of public engagement and active social networking, including the twitter hashtag #hospitalmicro. Westwood described the structure of the SIN, which includes 14 officers in the USA, to actively promote UK scientific expertise in different geographical regions. The specific aims of the SIN are to stimulate new scientific collaboration in strategic areas, strengthen UK innovation, inform policymaking and leadership, and influence science policy abroad. Westwood represents the Midwest US, which currently has ~ $7.4 Billion of US Federal research funding across 13 states [16], 79 million people, and represents a bigger economy than Germany. Westwood highlighted that AMR, and nosocomial infections generally, currently costs the US economy ~ $20 million in direct, and $35 million in indirect healthcare costs. The UK strategy for dealing with this problem highlights increased international collaboration.

Anthony Kessel (Director of Public Health Strategy at PHE) then presented ‘Beating the Superbugs’ which highlighted the interests and agenda of PHE. Kessel reviewed key moments in the history of infection control, from the miasma to germ theory of disease, including the roles of Antonie van Leeuwenhoek, Florence Nightingale, Louis Pasteur, and Robert Koch, leading to the 20th century revolution from transmission prevention and chemical sterilization to specific antimicrobial chemotherapy. In 1967 the US Surgeon General William H. Stewart (Public Health Service) apocryphally announced that the war on infectious diseases was won [17] despite the fact that Fleming had said as early as 1948 that microbial resistance to penicillin was inevitable. Kessel reminded us how rapidly things have changed, in that by 2011 a supposedly completely drug resistant form of tuberculosis was found. Kessel identified the key limitations behind the fight against AMR, including lack of novel antibiotics (only 1 new antibiotic has been released on the market in the last 2 years), inappropriate use of antibiotics, and rapid evolution of multi-drug resistant bacteria. One of the last resort antibiotic against such organisms, meropenem, is currently the 9th most used antibiotic in the UK. Kessel informed the workshop that of the 7 priority areas for dealing with AMR and nosocomial infections identified in the recent Department of Health led UK cross-government AMR strategy, 4 have been highlighted as being overseen by PHE including: 1. Optimizing prescribing practice (PHE-led; human health); 2. Improving infection prevention and control (PHE-led; human health); 3. Raising awareness and changing behavior (PHE-led; human health); 4. Improving the evidence base through surveillance (PHE-led; human health).

Jack Gilbert (University of Chicago, Argonne National Laboratory, USA) led the introduction of the Alfred P Sloan Foundation funded research program, the Hospital Microbiome Project, which leverages DNA sequencing of individual bacteria and bacterial/fungal communities in hospital environments to discover reservoirs of AMR and transmission routes for how bacteria move around the hospital environment. The HMP is currently sampling a private hospital, the Center for Care and Discovery, at the University of Chicago, which was accessible for ~5 weeks of sampling prior to any patients or medical professionals using the building. The project has been analyzing 10 patient rooms, 2 nursing stations and the human incumbents of these spaces either daily or weekly since Jan 15th 2013, and will finish this first round of analysis on January 14th 2014. Initial results have focused on exploring overlap between the microbial communities in different spaces, associated with different treatments for patient conditions, and have shown significant differences, suggesting that different patients and different treatment strategies leave specific microbial signatures in a space. Gilbert demonstrated the power of understanding the dynamics of the bacteria associated with different environments, especially humans, and the impact on understanding disease progression and treatment outcomes (e.g. [18]).

Benjamin C. Kirkup (Walter Reed Army Institute for Research, USA), co-PI of the HMP, discussed the complementary arms of the project outside Chicago as well as the larger need for hospital microbiome research. The Department of Defense manages one of the world’s largest healthcare systems. The 232 Military Treatment Facilities provide care to millions of patients, including the combat wounded, active duty soldiers, dependants and retirees. The military healthcare system takes an active interest in the health and welfare of all patients, with a special emphasis on those wounded in combat [19] and deployed ‘disease and non-battle injury’ (DNBI [20]). After the disclaimer, he led with a comment about the partnership between the UK and US militaries and their shared challenges as the result of asymmetric warfare, particularly blast injuries. Wound infections present serious sequelae of traumatic wounds from blasts or large caliber weapons. The infections occur in a minority of patients, but the measures to avoid infection impact all patients; debridements and amputations must be more conservative, tissue sparing less ambitious, and antibiotic administration more aggressive. Wound contamination is inevitable but epidemiology demonstrates that most infections derive from bacterial transmission within the healthcare environment. This is no fault of the providers; the patients are severely compromised and complete isolation is utterly impractical. At the same time, studies have demonstrated that both traditional infection control measures and hospital zoning can impact infection rates and character [21]. The current methods of study are dependent on culture methods [22,23] and are insufficient for effectively characterizing the microbiome of wound infections in clinical samples [24], the environmental microbiome in hospital samples [25-27], or the asymptomatic carriage of microbes by healthcare workers [28]. Because the art of clinical microbiology does not seek to represent the entire microbiome of the patient, but instead to select the pathogen like a needle from a haystack comparing molecular environmental surveys to culture-based patient assessments is inappropriate. Similarly, comparing molecular diagnostics in the patient to culture-based environmental surveillance or carriage statistics would be inappropriate, and correlations would be unlikely to generate meaningful results. The strength of clinical microbiology is the long-established correlations with clinical outcomes tailored in partnership with existing practices and available chemotherapies. This advantage is slowly being eroded as molecular methods enter the clinical laboratory and both associations and mechanisms are established linking molecular diagnostics to clinical outcomes [29,30]. One of the new outcomes from molecular investigations is a better sense of the temporal progression within healing and infected wounds. Systematic progression, ecological succession even, has been demonstrated in animal models [31] and potentially observed in patients (Kirkup, unpublished data). This kind of progression suggests an extended opportunity for migration of bacteria both in and out of the wound over time and suggests multiple opportunities for colonization and spread of both pathogenic and healing compatible microbiomes. This observation motivates one arm of the Hospital Microbiome Project, an effort to better understand the flow of organisms from a patient into a single hospital room over time, including the liminal spaces such as bandaging, clothing, bedding and medical devices in contact with the patient. An on-going study with samples from the Walter Reed National Military Medical Center is characterizing related samples in an observational fashion and bridging the gap between culture and molecular methods in the hospital environment. Kirkup also emphasized that the existing studies do not do justice to the complexity of the problem; that there is a need for further clinical studies, incorporating epidemiology into hospital ecology, and for basic science, both of which has been pioneered in the UK by researchers addressing specific pathogens such as *Clostridium difficile* and *Staphylococcus aureus*. He discussed specific needs for a sub-sequence type understanding of bacterial ecology as illustrated by antagonism diversity among closely related organisms, and the need to investigate gene expression, fitness and evolution in not only patient associated, but hospital associated environments. Detailed evolution and ecology in a hospital can only be fully resolved by experimentation outside of a hospital; experiments are needed with diverse bacteria, complex bacterial communities, and simulacra of hospital environments at diverse scales and degrees of fidelity. His most ambitious suggestion was the development of a national or international facility to house experimental hospital rooms with independent HVAC and plumbing; into which organisms could be introduced and through which they could be tracked.

Mark Pallen (University of Warwick, UK) provided insight into the long tradition of healthcare associated infection research in the UK, including John Pringles pioneering work on disease progression in hospitals in 1750; Florence Nightingale’s observations that infection took away more life than bullets in military conflict; and research by Gunn and Griffiths into cross-infection within hospitals. Indeed, potentially as a result of the pioneering and targeted approach that UK research has taken to tackle this problem, the cases of meticillin-resistant *Staphylococcus aureus* (MRSA) and *Clostridium difficile* in UK hospitals has significantly declined in recent years. Pallen highlighted the importance of the UKs national infrastructure, including the National Health Service (NHS), PHE, and Genomics England, in providing multi-disciplinary interaction and facilitating research. Indeed, research on microbial ecology and ecosystem dynamics is also excellent in the UK, which is essential if studies are to unpick the tangle of interactions in hospital environments that may lead to nosocomial infections. Pallen highlighted the power of ‘joined-up-thinking’, by demonstrating how the combination of experts in genomic epidemiology hospital infection and control, and microbial ecology, can be used to significantly improve patient outcomes in hospital environments.

Four representatives of major UK funding agencies and foundations then presented their portfolio of research and funding programmes to the workshop. Michael Ball from the Biotechnology and Biological Sciences Research Council (BBSRC) discussed work supported by metagenomic sequencing, as BBSRC do not explicitly fund medical research (which is primarily covered by the Medical Research Council (MRC)). Ball showed research programs funded by BBSRC that support the development of tools, such as metagenomics and bioinformatic analysis, which enable focus areas of research in food security, bioenergy and the basic biosciences underpinning health. The latter of these supports research providing evidence for health benefits in humans and animals. This includes work on aging and social activities that improve health, including work on the microbiome. BBSRC also focuses on data rich science, and synthetic biology. A recent BBSRC-funded expert working group led to the development of a responsive mode call for proposals that would support research that addresses resolving the technological and methodological gaps in metagenomics. Additionally, a network grant will shortly be awarded to build the community, and will help to focus the research agenda on improving metagenomic application and analysis.

Ghada Zoubiane (MRC) presented a summary on the MRCs current portfolio on infection and bacteriology, which supports ~ $500 million of research in this area, including public health and infection control. Ghada explained that funding is through four Research Boards (supporting project grants, programmes grants, centers and partnership grants), MRC institutes and units, fellowships and specific translational funding (Biomedical Catalysts/DPFS and Confidence in Concept). The MRC supported specific initiatives that are relevant to AMR. This includes the MRC Centre for Molecular Bacteriology and Infection (Imperial College London), the four UKCRC Translational Infection Research Initiative consortia, jointly funding and MRC-led, and two consortia supported as part of the MRC-Canada partnership on AMR. The MRC had major input into the Department of Health five-year strategy on AMR and is currently initiating discussions with other research councils and other major UK funders on future collaborations and coordination of AMR related activities. MRC is the UK representative at the European Joint Programming Initiative on AMR, which seeks to increase the value of national and EU research funding by joint planning, implementation and evaluation of national research programs.

Lara Bethke (The Wellcome Trust) introduced The Wellcome Trust, a global charitable foundation dedicated to achieving improvements in human and animal health. The Wellcome Trust has an endowment of around $27 billion USD and currently spends about $1.2 billion USD on charitable activities each year. The majority of their funding is either directly targeted to individual researchers (fellowships or Investigator Awards) or awarded for larger collaborative programmes (Strategic Awards), although the Wellcome Trust also offers a variety of other schemes that are tailored to particular fields or initiatives. They have historically supported work in medical microbiology through their general funding schemes. Most relevant to the Hospital Microbiome project, they have recently made three awards to UK researchers involving genomic approaches to diagnostic and public health microbiology. These awards were made through the Health Innovation Challenge Fund, which is a parallel funding partnership between the Wellcome Trust and the UK Department of Health to stimulate the creation of innovative healthcare products, technologies, and interventions and to facilitate their development.

Claire Kidgell (National Institute for Health Research) introduced the NIHR, which was established in 2006 to fund leading-edge NHS, social care and public health research. Kidgell highlighted that the NIHR health research system is comprised of four key components: i) management and information systems; ii) infrastructure; iii) faculty; iv) research programs. The NIHR specifically funds applied research with the capacity to improve patient and public health outcomes. In 2013, NIHR issued a themed call for proposals is focused on AMR, and was launched in direct response to the publication of the 2nd volume of the 2011 Annual Report of the Chief Medical Officer: Infections and the rise of antimicrobial resistance with the goal of reducing the development and spread of AMR [32]. Research in this area may encompass better prevention and improved surveillance.

### Session 2: Discussion of potential UK funding and value of the UK to such research

#### Moderated by Mark Pallen

Pallen led a lively discussion focused on the best way to secure UK funding to tackle AMR, the potential to use new technologies to explore the microbiome in health care environments, and the value to the UK of such research. A specific outcome of this discussion was that the UK’s infrastructure of NHS trusts, combined with its extensive and successful work in reducing the rate of health care associated infections makes it an attractive and productive option for implementing extensive studies of microbial ecology in hospitals. Mark Wilcox pointed out that there is no point simply repeating what’s already done in the US in a UK hospital; therefore any experiments performed on the hospital microbiome in the UK must be translational. Alison Holmes suggested that the microbiome analysis in the existing network of UK hospitals would provide opportunity for working in many different clinical spaces, with networks of staffing that can be manipulated to derive translational affects. Peter Wilson highlighted existing work on tracking *Clostridium difficile* infections and spores on surfaces in hospitals; this information is being used already to augment cleaning strategies and identify novel reservoirs of this specific organism. Julian Parkhill highlighted the extensive research in UK hospitals on near-real-time tracking of pathogen genomes in hospital systems, and Derrick Crook has shown that most *Clostridium difficile* cases originate from infections brought into the hospital, not from internal reservoirs [24]. This suggests that 16S rRNA amplicon and shotgun metagenomic tracking of microbial communities may not identify reservoirs for key nosocomial infections, and also suggests that looking for reservoirs in the hospital might not be an appropriate experimental approach. Beryl Oppenheim pointed out that most of the existing research and observational work has focused on symptomatic, not asymptomatic, patients, therefore we don’t know the role of asymptomatic patients as conduits for pathogen transfer, let alone the environment as a transient reservoir. Jack Gilbert pointed out that if a non-pathogenic strain passes through an asymptomatic patient, it might become pathogenic following interactions with their host microbiome or human-cells (e.g. [33-37]). Therefore screening large numbers of patients and surfaces at daily resolution may enable the identification of key events of pathogenization of these organisms, which could then be used to design specific validating experiments.

The discussion then moved on to what information exists on staff carrying potentially pathogenic organisms, and while frameworks for dealing with staff with MRSA, *Clostridium difficile*, etc. do exist, we have no data on whether staff sickness events (even for viruses, antibiotic use, etc.) correlate with onset of nosocomial outbreaks. Mark Wilcox suggested that staff could also be a reservoir, and importantly, while we often know about classical pathogens and what their distribution is in a given population, we know virtually nothing about Gram-negative bacterial infections in these cohorts. Indeed a lot of Gram-negative multidrug-resistant bacteria are being identified, and these appear to be isolated in specific sites in hospitals, e.g. Pseudomonas spp in neonatal wards [38]. There is an immediate need in any population to move beyond the classification of the major diseases, e.g. MRSA and *Clostridium difficile*, and to expand into other nosocomial groups such as novel Gram-negative strains, but also unknowns. Hence surveys can play a role in uncovering new potential pathogens. Gilbert suggested that exploring the microbial community could be very important for predicting which people or buildings have the potential to become infected or colonized by a given organism. Mark Wilcox suggested that this was indeed the case, and the potential for susceptibility to be directed by the state of the microbiome is absolutely biologically plausible, however, the application of this at a scale relevant to hospital patient turnover was not technically feasible. Wilcox suggested that longitudinal studies were essential to get at these questions, and hence the existing Hospital Microbiome Project had considerable potential. However, he pointed out that to get relevant information it is essential to focus these longitudinal studies on high-intensity spatial analysis, focused on a small site, compared to spreading the research sampling too thinly.

Mark Pallen explored the translational potential of microbiome analysis, e.g. even if we were able to identify each disease in a system, how would we change hospital architecture or operation to reduce infection rates, e.g. do we isolate people? Develop specific strategies for patient room rotations? Wilcox suggested the need to uncover how to intervene in disease colonization and progression, rather than ‘cleaning-up the mess’ afterwards. Therefore, any efforts to characterize the microbial profiles associated with nosocomial outbreak susceptibility/infection should be designed to target mechanistic steps in infection that can be interrupted. Pallen highlighted that this was key; i.e. why do pathogens colonize certain people, and how do we stop them from being infected? He suggested it might be possible to screen every patient coming into the hospital to characterize their microbiome, pathogen load, and determine how they will respond to a given treatment. This may also be used to drive zoning of patients within hospital buildings, which could also influence building design.

Danny Wilson highlighted the power of genomics, which has now been provided with an incredible breadth of analysis; however, currently the application of metagenomics provides much less resolution than targeted whole genome sequencing for detecting person-to-person transmission. Gilbert disagreed about the degree of resolution lost by an inability to assemble genomes. He claimed that from metagenomics, one could determine which organism and functional types exist in a space, and how the specific assemblage may interact with known pathogens. Gilbert also suggested a correlative function for metagenomic data; that the identification of a metagenomic fingerprint with an absence of outbreak activity would be indicative of a ‘healthy’ community that could be used to reduce risk of infection in those people or spaces with a depleted microbiome, i.e. a ‘healthy probiotic’. Pallen highlighted that if we want to use probiotics to improve health in buildings for people, then we need to know that the bacteria we are adding can never become pathogenic, however, this is a very conservative approach and probably technically impossible as bacteria can always evolve. Gilbert pointed out that clinicians already use simple probiotics to augment health in those that have taken antibiotics, and also to reduce risk of infection from other pathogens.

From an architectural perspective, Peter Wilson highlighted building materials as being key elements in determining survival of microbes associated with a given surface, as well as proliferation. Certain materials have a much higher transmissible potential, however, this is currently poorly characterized. We know there are high touch surfaces that are recognized transmission centers, and cleaning these has been shown to reduce the microbial density, but there is very little information regarding whether this affects clinical symptoms in patients. Phil Marsh suggested that different cleaning strategies for different surface should be validated; there is far too little data on how each cleaning strategy reduces infection risk. Jimmy Walker highlighted the need for water surveillance especially for key pathogens such as *Legionella*. Wilcox pointed out that there are extensive surveillance projects out there, but they are not evenly distributed across different hospitals, or even within a given hospital, which limits their effectiveness for interpretation.

Marsh suggested that funding for characterizing the general health promoting aspects of bacteria, i.e. not looking at disease causing organisms, was virtually impossible given the current funding climate. The MRC and WT highlighted that their organizations welcome research to promote health and wellbeing and are not restricted to disease-focused projects.

In wrapping up this session, Gilbert suggested that while it is no panacea, intense sampling and characterization of the longitudinal and biogeographic distribution and dynamics of the microbiota could help define predictive models, especially when combined with infection control and immunity data, patient records, and building science measurements. These models could be used to predict events in other hospitals, which would then need to be validated. Peter Wilson suggested that individual genomes couldn’t be continuously surveilled, therefore the 16S rRNA amplicon sequencing could be used to target the environments in which you might like to perform near-real-time genome sequencing. Julian Parkhill asked a pertinent question, as to whether big-data, i.e. massive characterization of a hospital environment could actually help to predict disease outbreaks and identify mechanisms of infection and transmission. Gilbert suggested it could, but that this would need to be proven. Parkhill suggested that it would be essential then to take a coordinated approach to analyzing the microbiota in the built environment. However, determining which disciplines to coordinate with would be the key to future projects. Some examples given were, surgeons, clinical microbiologists, infection control and immunity, nurse teams, cleaning staff, etc.

### Session 4: Highlighting the potential issues for AMR and microbiome research

#### Moderated by Dawn Field and Julian Parkhill

Session 3 tackled some aspects regarding the design of microbiome experiments specifically to address potential translational elements into disease transmission characterization in the hospital systems. The UK has much to offer, and much to gain from such work. Intense discussion focused on existing differences in UK and US methods for tackling AMR, especially regarding experimental design. This also highlighted the differences in scale and scope of project where the main focus is to understand complex community dynamics versus a single known pathogen (i.e. MRSA). Discussions revealed that there is not yet a consensus on the impact that the built environment microbiome has on disease transmission in hospitals nor on the effect of different types of surfaces. In fact it is possible that no such relationship exists. However, it is also possible that such a scientific foundation may be required to help design future experiments to test these hypotheses.

Parkhill started the discussion by highlighting that there is the room to use genomic technologies to explore microbial community distribution, especially pathogens, in the built environment, and this is something that could be extensively explored in the UK because of its history of very detailed epidemiological, reductionist focus on the spread of specific, major pathogens. However, there is extensive work that would need to be done before it could be said to fulfill the specific translational needs highlighted by the UK research councils. Much of the clinical research agenda is driven by the need to implement evidence-based approaches in clinical practice, and stronger evidence of the value of microbiota studies in this arena would be needed to convince the funding agencies to follow this avenue of research.

Peter Hoffman brought up a significant concern with microbial community profiling, specifically whether the organisms identified are active or even transmissible, i.e. just because a 16S rRNA sequence is present doesn’t mean the corresponding bacterium is alive or infectious. Also, in all these sampling efforts it is essential to distinguish between wet and dry environments; in dry environments the microbes are probably not active and could form contamination of a transient nature, but in wet environments microbes are more than likely actively interacting. Therefore, if the microbiome had the potential to influence pathogen dispersal or to determine whether an organism could cause infection, it would more than likely occur in the wet regions of a building. Microbial contamination that lands in dry areas tends to dry out too quickly for the microbes within it to replicate, but Beryl Oppenheim suggested that just because an organism has dried out on a surface, it does not mean that it is dead – should the surface be re-wetted certain microorganisms might become replicate again. Peter Hoffman also suggested it is very important that we define the terms ‘contamination’ and ‘colonization’; in contamination the organisms will not replicate and, once removed by cleaning, will not re-establish; in colonization the organisms are persistent in that niche. Persistence suggests the capacity for growth, and water is very important in this.

Parkhill suggested that correlative studies must always be treated with care, for example, just because you have *Legionella* in the water supply, and a *Legionella* outbreak occurs in the hospital, it doesn’t necessarily mean that these two facts are linked. To prove these instances would require improved experimental design and resolution of analysis. Microbiome analysis may not be able to address this concern, but it is a powerful tool for suggesting a relationship that could be tested.

Dawn Field encapsulated the 3 branches of analysis being discussed that could influence understanding of AMR and nosocomial distribution through hospital environments. First, genome analysis of individual organisms associated with disease events; second, population dynamics of pathogens within the building and patient population; finally, microbial ecology, which probably influences the previous avenues, and could help identify novel pathogens, e.g. multidrug resistant Gram-negative bacteria.

Wilcox suggested that instead of implementing “discovery-focused” global microbiome analysis in the UK hospital systems we should instead build on existing strengths. For example, the UK has historically had more success in controlling antimicrobial prescription and application. In the US, due to the broader private healthcare system, and lack of a central doctrine, control of antibiotic use is more problematic. For translation potential, control of antibiotic application is probably the most effective means to have rapid success. Field interjected that this would require significant investigation of the impact of human antibiotic use in medicine and the food industry on environmental reservoirs, and their impact on healthcare. However, it will not deal with all nosocomial infections, and fails to uncover the mechanisms associated with the development of new diseases. Gilbert highlighted this by suggesting the potential of metagenomics to identify and track the antibiotic resistance of bacteria that we don’t normally track, e.g. non-pathogens, which could have the potential to transfer such resistance to pathogens.

Wilcox discussed how to design experiments, and pointed out that it is currently impossible to do a statistical power calculation to design specific experiments. Derrick Crook suggested that this was a major issue with microbiome research, in that observational strategies could never be used effectively to answer these questions, just to pose more questions. Crook pointed out that we don’t even know how to define disease state properly within a patient, let alone how to correlate disease state with changes in the microbiome of different patients, and even further complications from the microbiome of building spaces generates too many confounding factors to make such data useful. This limited even the possibility to define hypotheses from these data. However, the investigation space is vast, and these tools can help to contextualize the spaces in which we can and cannot make such progress.

Overall, the session revolved around the complications of observational correlative analyses, and especially the limitations. It was suggested that the funding climate within the USA was more conducive to supporting the exploration of whether the microbiome analysis techniques could be used to make progress on the problem of clinical AMR and nosocomial infections.

### Session 4: Highlighting the potential issues for AMR and microbiome research

Immediately prior to the wrap up and summary, Gilbert chaired a session aimed at identifying potential focused topics and areas of concern that must be dealt with when considering AMR research, especially when examining the microbiome. Fourteen areas of interest were identified by the workshop as being worthy of further discussion, with the aim of developing these ideas in a position paper.

(1). Public and professional engagement – the need to have better communication between research scientists and the public and clinical professionals was identified as important. Explaining the key problems associated with AMR research, and the necessity for exploring novel analytical strategies such as targeted microbiome research, is difficult, and requires training to make any messages effective.

(2). Pre-illness primary care analysis – a need for understanding the ‘healthy’ microbiome of people was identified as a key issue, not just as a test case (e.g. NIH Human Microbiome Project), but in a controlled clinical study to determine key biomarkers associated with disease onset and/or intra-hospital transmission events. Examining the patient microbiome both before and after hospitalization was suggested as important research avenue.

(3). Global health strategy impacts – it was considered highly important that any research performed under these auspices be held up against a need to impact global health strategies, i.e. not just those in the USA and or UK. It would be very important therefore to determine how microbiome studies could be effectively explored in developing countries.

(4). Assessing cleaning strategies in hospitals – it was considered vitally important that more research be focused on the value and impact of different cleaning strategies in hospitals. It was especially important to explore inappropriate claims regarding different strategies, and limit expectations for novel ‘breakthroughs’. Cleaning contains a major sociological factor that must be considered, such as ‘pride in work’, and appropriate pay, training and education for staff.

(5). Examining contribution of water versus air contamination – the source and reservoirs of nosocomial infections is particularly important. Performing controlled experiments to examine the relative contributions of the reservoirs in air and water is essential; the majority of existing research focuses on these separately, yet they directly and indirectly interact.

(6). Antibiotic exposure – the culture of antibiotic use must be far better catalogued (especially in the USA) so that trends can be understood, and where necessary, altered. This is essential to inform genomic and metagenomic databases, so as to define hospitals based on their antibiotic administration culture. This may help identify new ways in which we can alter treatment strategies towards improved patient outcome.

(7). Impact of immune suppression on nosocomial spread – to determine how these cohorts can influence the spread and development of AMR and nosocomial infections it is vital that these easy to access cohorts are used for directed clinical trials. Understanding whether their microbiota plays a role in AMR and nosocomial spread will require multidisciplinary research activities.

(8). Impact of illness frequency and disease state in staff – difficulties with data acquisition due to fear over job security, has led to limited information on whether nosocomial infections or AMR has a reservoir in staff. Most staff do not report in for work when they are ill, however, records need to be improved if we are to rule out this as a source.

(9). Gram-negative bacterial infections – one of the understudied hospital infection types are those bloodstream and critical care infections caused by Gram-negative bacteria. The workshop identified this as a vital area from improved research into the ecology and transmission in health care environments. For example, we know next to nothing about their origin and mode of transmission, especially when compared to ‘*Clostridium difficile*’ and MRSA.

(10). The impact of hospital ‘type’ on infection risk – recent evidence has suggested that private health care establishments in the UK have a higher level of AMR than NHS facilities, and while this evidence is circumstantial, it suggests that the management practices for infection control, combined with the differences in patient populations (e.g. more foreign national patients in private healthcare), could have an impact on nosocomial rates.

(11). Healthcare-associated architectural and technological design – the need to consider the influence of building and healthcare product design on the development and spread of AMR was specific highlighted. Particularly the need to interact with and educate architects and product designers, and co-develop experimental evidence to support specific claims for key improvements.

(12). Development of metagenomic methodologies – as highlighted by BBSRC, there is a vital need to develop quantitative metagenomic tools to better investigate microbial interactions that lead to the development of AMR and pathogenicity. More rigorous investigation of these tools, including their limits, is needed to rely on them as a source of valuable information.

(13). Understanding the impact of food and drink provision in hospital environments – food and drink, as well as general ‘alternative’ sources of microbial contamination in hospitals need to be more rigorously investigated. Many catering firms for hospitals are regulated, but the aspects of regulation that may lead to a reduction in the development of AMR and nosocomial infections associated with catering services is not fully understood

(14). The role of selective decontamination – investigating decontamination strategies that need to be implemented when AMR pathogens are identified need to be continuously re-evaluated, especially with the potential of metagenomic methods to identify novel bacteria, fungi and viruses, which could be a cause of diseases for which a known trigger has not yet been elucidated. Such novel taxa may have very different responses to existing decontamination methods.

While far from exhaustive, this list highlights a number of key areas that need to be considered, and for which existing data is inadequate. As always, the research community associated with this area is constantly exploring new avenues, which exemplifies the need for blue skies research paradigms.

### Wrap-up and summary session

Consensus amongst the group was that microbiota are likely to play a role in causing disease but there was considerable debate over whether a greater understanding of microbial communities in hospitals could provide transformational advances in our understanding of the origins, spread, and persistence of AMR, or could lead to better targeted interventions. Extensive Twitter coverage with participants encouraged to tweet using the hashtag #hospitalmicro generated a total of 82 original tweets and 58 retweets with a total reach of 47,033 users, which highlights the reach of this meeting.

This was an interesting and worthwhile meeting that may influence how AMR research is approached in the UK. It was interesting to observe the divide between existing US and UK approaches in this area, and the skepticism of some of the UK representation on the immediate value of conducting HMP-type studies in UK hospitals. This was in part due to the more explicitly translational agenda of UK funding agencies in the clinical arena, compared to the willingness of US philanthropic foundations to generously support built environment microbiome studies, which when combined with federal support from agencies such as the National Institute for Health, currently stands at ~ $2.4bn. Implantation of HMP studies in the UK has the potential to be fruitful in increasing our understanding of hospital-associated infections and could direct targeted intervention strategies. However, in the current UK funding climate, such studies would likely have to be more clearly translational to be successfully funded.

### Evening reception

An evening reception at the FCO included 35 additional guests from government, industry, and the press, who joined the workshop group to continue discussions, and broaden interest in multiple AMR prevention strategies. The group was joined by Prof John Watson (Department of Health Deputy Chief Medical Officer), and Andrew Jackson (Head of the UK Science and Innovation Network and FCO Deputy Chief Scientific Advisor). Prof Watson and Mr. Jackson addressed the group along with Dr Gilbert, and emphasized the role of SIN in helping to facilitate events such as these, the international aspects of DH’s AMR strategy, and the outcomes of the workshops discussions. In general it was agreed that human health and pathogens are a global issue, and countries should be working together to develop a future in which community level discovery and reductionist level pathogen genomics coordinate to create a knowledgebase to help facilitate future work.
